# Isolated Spinal Metastasis with Spinal Cord Compression Leads to a Diagnosis of a Follicular Thyroid Carcinoma

**DOI:** 10.7759/cureus.346

**Published:** 2015-10-12

**Authors:** Gentian Toshkezi, Michael Galgano, Silva Libohova, Satya Marawar

**Affiliations:** 1 Neurosurgery, SUNY Upstate Medical University; 2 Bachelor of Science, Temple University; 3 Orthopedic Spine Department, Syracuse Veteran's Affairs Hospital

**Keywords:** thyroid follicular carcinoma, spine metastasis, back pain, radioactive iodine ablation

## Abstract

Introduction: Thyroid carcinoma initially presents with clinical symptoms due to metastatic lesions in less than 5% of cases. Spinal cord compression from an epidural metastatic lesion as a first symptom is extremely rare. One would expect such a presentation to occur much later in the course of the disease.

Methods: We are presenting a case report of a follicular thyroid carcinoma that presented with spinal cord compression from a thoracic epidural metastatic lesion in a previously healthy 55-year-old male. A single metastasis of follicular thyroid carcinoma presenting with posterior spinal cord compression is rare. In this particular case, our management included a mid-thoracic laminectomy, followed by resection of the epidural lesion. Once the surgical pathology confirmed the diagnosis of a follicular thyroid carcinoma, the general surgery team performed a near total thyroidectomy, after which he received radioactive iodine therapy. The patient is symptom-free at his three-year follow-up.

Conclusion: Initial presentation of follicular thyroid carcinoma with symptomatic thoracic myelopathy from an epidural metastasis is very uncommon. An early diagnosis and prompt surgical intervention provided an excellent outcome.

## Introduction

Thyroid carcinoma initially presents with clinical symptoms due to metastatic lesions in less than 5% of cases. Spinal cord compression from an epidural metastatic lesion as a first symptom is extremely rare. One would expect such a presentation to occur much later in the course of the disease. Thyroid cancer is rare and accounts for roughly 1% of all new malignant disease with a male to female ratio of 1:3. Thyroid follicular cancer is found more often in areas of endemic goiters, and iodine deficiency has been reported as an independent risk factor [1]. Follicular carcinoma metastasizes to bone in 2-13% of the patients. Spinal metastases most commonly involve the vertebral body and can lead to pathologic compression fractures and instability [2]. The spine is actually the most common bony metastatic site in thyroid carcinoma [3].

## Case presentation

Informed patient was consent was obtained for this patient's treatment.

**Figure 1:**
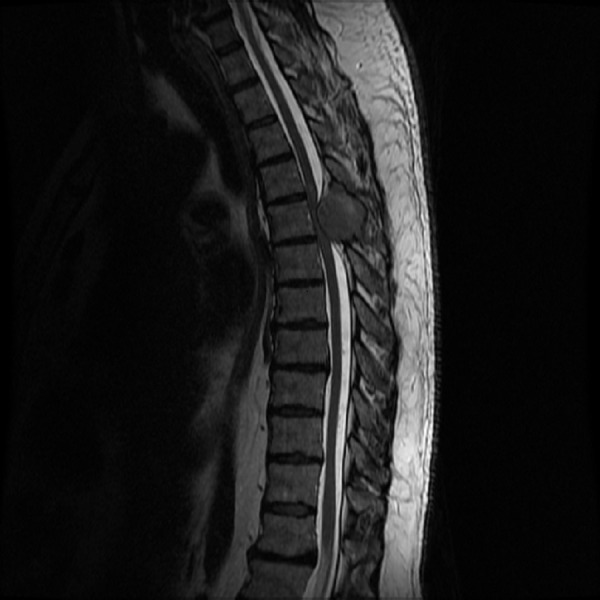
Preoperative T2W sagittal images. MRI of the thoracic spine T2W sagittal images revealed an approximately 2.9 x 3.8 x 3.7 cm extradural midline mass centered in the posterior elements of T5 from T3-T4 to T6-T7. The mass extended into the spinal canal with severe thoracic cord compression. There was increased signal within the spinal cord from the lower T4 level to the upper T6 level consistent with edema. No extension into the neural foramina was noted.

**Figure 2:**
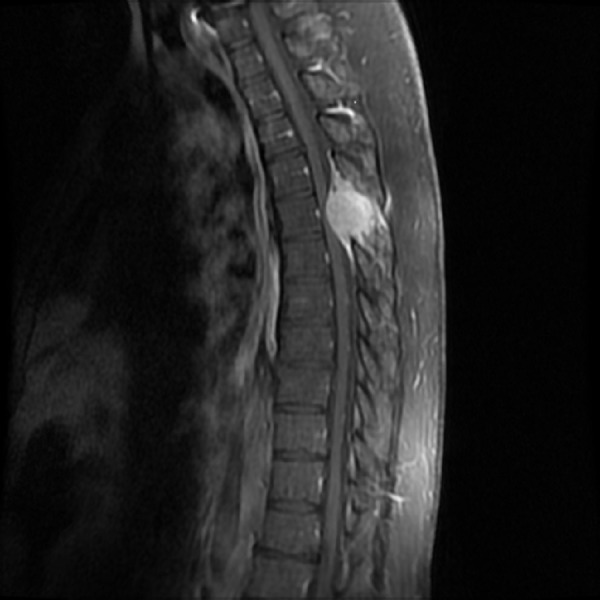
Preoperative Short TI Inversion Recovery (STIR) sagittal images. MRI of the thoracic spine STIR sagittal images revealed an extradural midline mass centered in the posterior elements of T5. The mass was extending into the spinal canal, with severe thoracic cord compression.

**Figure 3:**
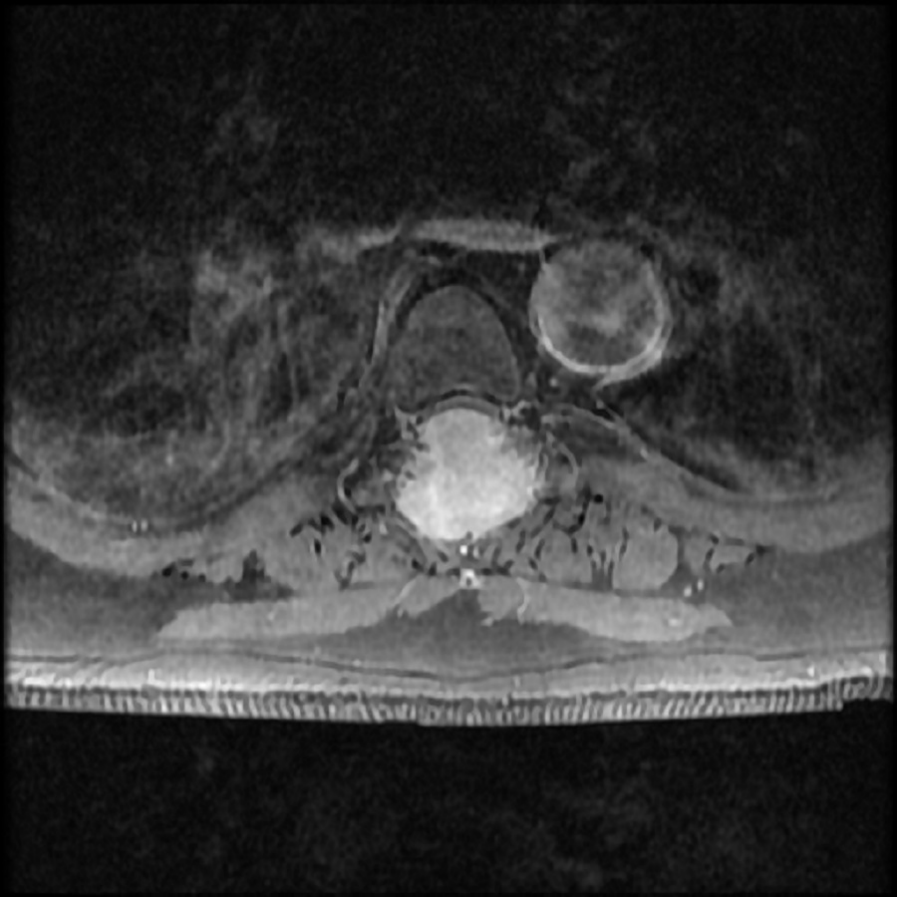
T1 axial images with contrast MRI of the thoracic spine at T1 with contrast axial images revealed an approximately 2.9 x 3.8 x 3.7 cm extradural midline mass centered in the posterior elements of T5. The mass was extending into the spinal canal with severe thoracic cord compression. There was increased signal within the spinal cord.

We present a case of a 55-year-old male with no significant past medical history, who presented to emergency department with a recent onset of gait imbalance, as well as subjective numbness and hypoesthesia of the bilateral lower extremities. He had sustained multiple falls during the previous two weeks prior to admission. Shortly after admission, he developed urinary retention. An MRI of the thoracic spine revealed an approximately 2.9 x 3.8 x 3.7 cm extradural midline mass centered in the posterior elements of T5 (Figures 1-3).

The mass extended into the spinal canal with severe thoracic cord compression. There was increased signal within the spinal cord from the lower T4 level to the upper T6 level consistent with edema. The enhancement pattern was fairly homogeneous, with extradural enhancement extending superiorly to the T3-4 level and inferiorly to the T6-T7 level. No extension into the neural foramina was noted. The subsequent radiographic and metastatic workup was negative for a primary malignancy or any other metastatic lesions. Over the course of the first twelve hours of admission, the patient’s neurological exam continued to decline with a deterioration of his sensory symptoms. He was then given 10 mg of intravenous dexamethasone and surgical planning was undertaken.

Hospitalization:  The patient was taken to the OR under general anesthesia. He was positioned prone on the Jackson table. Under fluoroscopy, we identified the level of the T5 spinous process, counting proximally from the sacrum. The incision was made over the spinous processes from T4-T6. The fascia was incised on both sides of the spinous processes. We performed a subperiosteal muscle dissection on both sides simultaneously. The tumor was readily visible anteriorly to the spinous process of T5, which was partly involved in the tumor. We dissected around the tumor with good visualization of bone lateral to the tumor with exposure of the facets. The facets were predominantly intact on both sides. The lamina of T4 and T6 were further exposed. A piece of the tumor was sent for frozen section and received a diagnosis of metastatic carcinoma. We then performed laminectomies at T4 and T6. There appeared to be a clear plane between the tumor and the dura. The tumor was then dissected from the dura fairly easily, and from the bony edge of the T4-5 facets. We were able to perform an en bloc resection of the tumor while preserving the facet joints on both sides. After removal of the tumor, the dura was intact. Complete resection was confirmed. Hemostasis was undertaken, and the dura appeared to be well decompressed. Closure in layers was performed.

Pathology results were consistent with a metastatic thyroid carcinoma, follicular type, with focal vascular invasion, extending to the bony borders. Immunostain (TTF-1 positive, thyroglobulin weakly positive, calcitonin negative, and CEA negative) aided in confirmation. 

**Figure 4:**
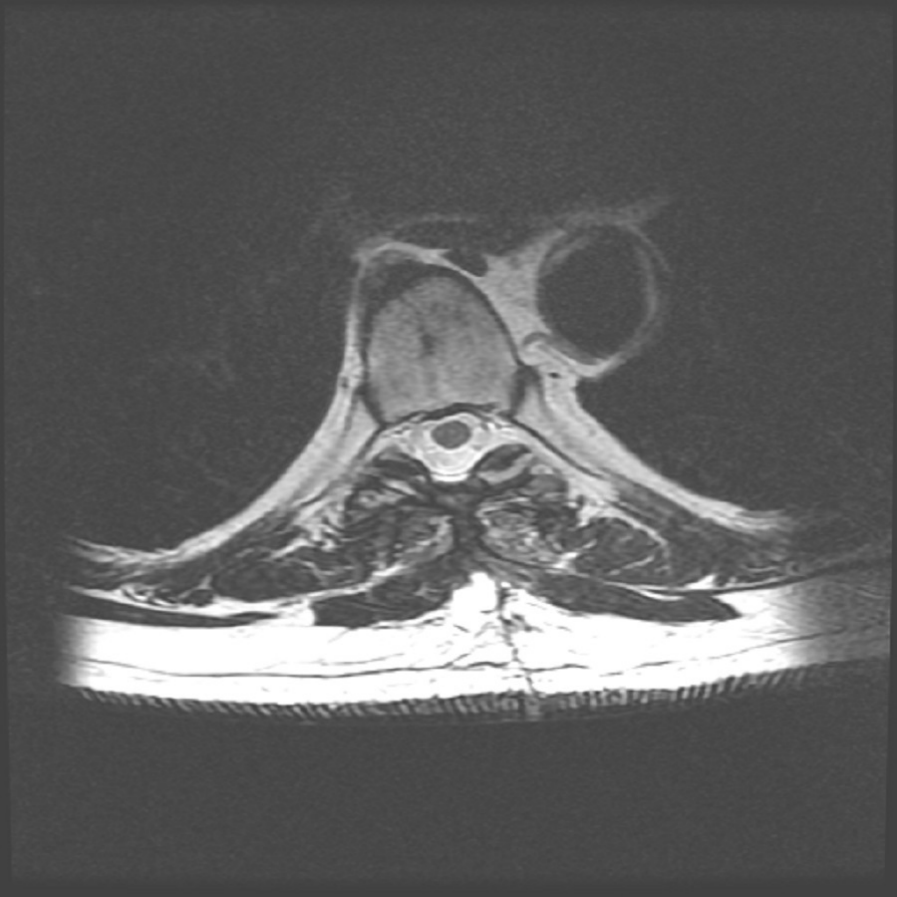
Postoperative T2 axial images. T2-weighted axial images of the thoracic spine MRI shows postoperative changes with en bloc resection of the epidural tumor with decompression of the spinal cord.

**Figure 5:**
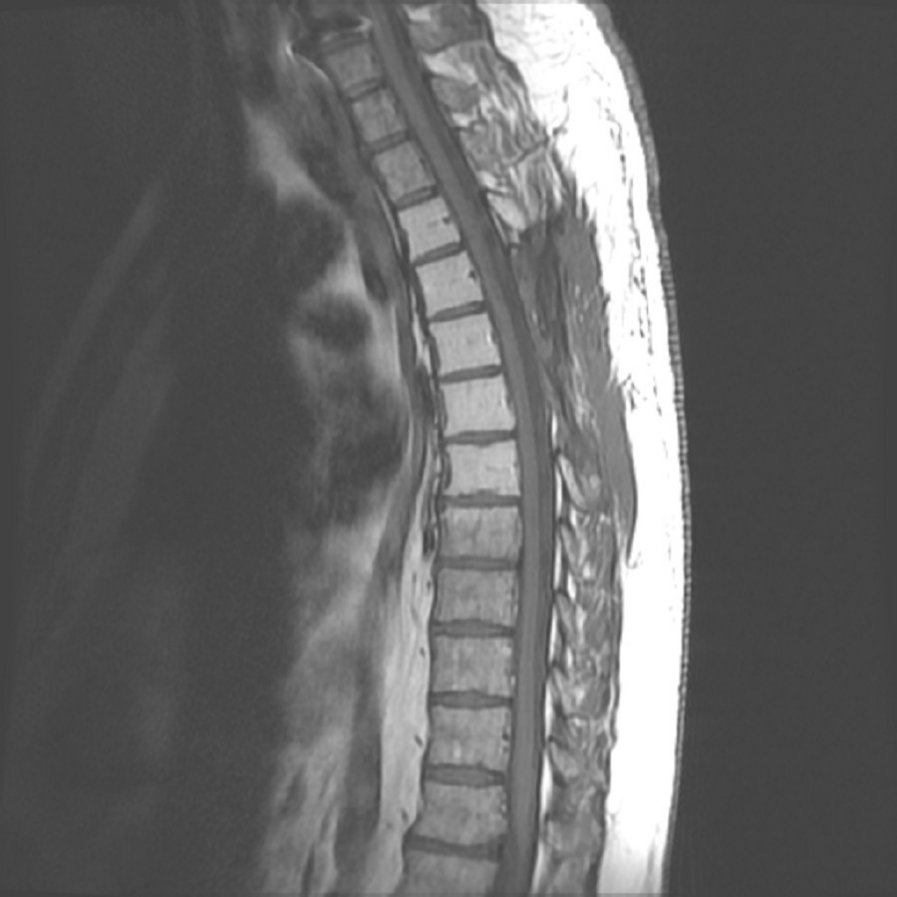
Postoperative T1-weighted images with contrast. T1-weighted sagittal images with gadolinium of the thoracic spine MRI show postoperative changes with no evidence of a residual mass at the T4-T6 levels. There is a decompression of the spinal cord.

The postoperative course was uneventful. A postoperative MRI of the thoracic spine showed satisfactory resection of the epidural tumor with decompression of the spinal cord (Figures 4-5). 

After the operation, the patient fully recovered from his sensory deficit, had improved gait, and regained normal urinary bladder function.

A thyroid ultrasound was performed. The right thyroid lobe measured 4.8 x 2.1 x 1.4 cm. It contained a tiny hypoechoic upper pole nodule measuring 4 x 3 x 2 mm. The left thyroid lobe measured 3.8 x 2.5 x 1.9 cm. It contained a dominant, heterogeneous, solid-appearing upper pole nodule measuring 2.0 x 1.8 x 2.0 cm. After an extended metastatic workup with no other metastases, a near-total thyroidectomy was performed. The patient is currently doing well without any particular symptoms. 

## Discussion

Thyroid cancer is rare and accounts for roughly 1% of all new malignant disease with a male to female ratio of 1:3. Thyroid follicular cancer is found more often in areas of endemic goiters, and iodine deficiency has been reported as an independent risk factor [1]. The addition of iodine in salt likely explains the decrease in follicular thyroid cancer, although there is an increase of the papillary type. Activation of an oncogene in thyroid follicular carcinoma is common with the rat sarcoma (RAS) gene mutation found in 80% of follicular carcinoma [4].

The follicular type tends to typically affect middle-aged females. In 90% of the patients, it presents as a simple thyroid nodule. A distant metastasis is the first manifestation in only 5%. The most common sites of metastases are the bone, lungs, and lymph nodes [5]. Follicular carcinoma metastasizes to the bone in 2-13% of the patients. Spinal metastases most commonly involve the vertebral body and can lead to pathologic compression fractures and instability [2]. The spine is actually the most common bony metastatic site in thyroid carcinoma [3].

From all thyroid cancers, Hurthle cell carcinomas have the highest rate of spinal metastasis at 12%. Spinal metastases are more frequent in the thoracic spine (60-80%), followed by the lumbar spine (15-30%) and cervical spine (< 10%). The metastatic spread is achieved either through Batson plexus, direct spread from local invasion, or less commonly through cerebrospinal fluid pathways. The spine metastases of thyroid cancer have a predilection for the vertebral body. The clinical manifestations most commonly encountered are related to pathological vertebral body collapse or instability, and often manifest as back pain. Radicular pain may also be a clinical manifestation depending on the tumor infiltration pattern. Spinal cord compression is more commonly seen ventrally due to an extension of a vertebral body tumor into the spinal canal [6].

Prognosis of thyroid carcinoma with spinal metastases is more favorable compared to other spinal metastatic cancers. The 10-year survival in a patient with a pulmonary metastatic disease of follicular thyroid cancer after surgery and radioactive iodine ablation is up to 90% and curative in 35-40%. Bone metastasis has a worse prognosis than pulmonary ones because of lower concentration of the radioactive iodine and requires higher doses. Ten-year survival in patients with bone metastasis is 12% and 8% for 20-year survival [3].

Spinal metastases from a thyroid malignancy usually present with a more insidious onset. There have only been a few other reported cases of symptomatic follicular thyroid metastatic disease to the spine presenting with neurological compromise [3-5, 7-12]. The case we present here is unique as the patient’s first clinical manifestation was thoracic myelopathy, and it was caused by a spinal epidural metastasis with dorsal spinal cord compression from an undiagnosed follicular thyroid cancer. The onset was rather abrupt and progressed quickly over a two-week period. The location of the lesion we encountered was in the dorsal elements, which is very atypical for thyroid metastases to the spine. The treatment was urgent decompression and en bloc tumor resection. The postoperative course was uneventful, and the patient underwent further testing with a thyroid ultrasound and consequently resection of the thyroid nodule. Further treatment options for patients with follicular thyroid carcinoma include radioactive iodine ablation, whole body imaging, and a thyrotropin suppressive dose of thyroid hormone. Patients with positive lymph nodes may require compartment-oriented neck dissection [1].

## Conclusions

The case we have presented is quite rare since thyroid metastatic lesions do tend to predominate in the vertebral bodies, as opposed to the epidural space, as it did in our patient.

Through this case report, we want to emphasize that thyroid cancer is a possibility in the differential diagnosis in patient presenting with spinal cord compression from a posterior mass lesion. Early diagnosis and prompt treatment of symptomatic spinal metastatic thyroid cancer provides optimal results in recovery and extending the life expectancy. Optimal management entails obtaining the proper radiographic studies, such as a contrasted MRI, as well as prompt surgical treatment when necessary. Thyroidectomy with radioactive iodine ablation increases the 10-year survival in patients with metastatic disease.
